# Dietary Intake as a Link between Obesity, Systemic Inflammation, and the Assumption of Multiple Cardiovascular and Antidiabetic Drugs in Renal Transplant Recipients

**DOI:** 10.1155/2013/363728

**Published:** 2013-07-30

**Authors:** Bruna Guida, Mauro Cataldi, Immacolata Daniela Maresca, Roberta Germanò, Rossella Trio, Anna Maria Nastasi, Stefano Federico, Andrea Memoli, Luca Apicella, Bruno Memoli, Massimo Sabbatini

**Affiliations:** ^1^Department of Clinical Medicine and Surgery, Physiology Nutrition Unit, University Federico II of Naples, Via Sergio Pansini 5, 80131 Naples, Italy; ^2^Department of Neuroscience, Reproductive and Odontostomatological Sciences, University Federico II of Naples, Naples, Italy; ^3^Department of Public Health, Nephrology Unit, University Federico II of Naples, 80131 Naples, Italy

## Abstract

We evaluated dietary intake and nutritional-inflammation status in ninety-six renal transplant recipients, 7.2 ± 5.0 years after transplantation. Patients were classified as normoweight (NW), overweight (OW), and obese (OB), if their body mass index was between 18.5 and 24.9, 25.0 and 29.9, and ≥30 kg/m^2^, respectively. Food composition tables were used to estimate nutrient intakes. The values obtained were compared with those recommended in current nutritional guidelines. 52% of the patients were NW, 29% were OW, and 19% were OB. Total energy, fat, and dietary n-6 PUFAs intake was higher in OB than in NW. IL-6 and hs-CRP were higher in OB than in NW. The prevalence of multidrug regimen was higher in OB. In all patients, total energy, protein, saturated fatty acids, and sodium intake were higher than guideline recommendations. On the contrary, the intake of unsaturated and n-6 and n-3 polyunsaturated fatty acids and fiber was lower than recommended. In conclusion, the prevalence of obesity was high in our patients, and it was associated with inflammation and the assumption of multiple cardiovascular and antidiabetic drugs. Dietary intake did not meet nutritional recommendations in all patients, especially in obese ones, highlighting the need of a long-term nutritional support in renal transplant recipients.

## 1. Introduction

The outcome of a successful renal transplantation (RT) depends on immunological and nonimmunological factors that may lead to progressive graft damage and, ultimately, to chronic allograft dysfunction (CAD). Besides a valid clinical assessment and a balanced immunosuppressive therapy, patients compliance to a correct lifestyle may help preventing CAD. In particular, healthy eating habits in accordance with current dietary recommendations represent an effective secondary prevention strategy that reduces the risk of renal and cardiovascular diseases [1–7]. This is particularly important in renal transplant recipients [7–9]. In these subjects, an increase in body weight commonly occurs. Several factors are responsible for this phenomenon; among them the most relevant are the transition from restricted dialysis dietary regimen to unrestricted diet, the feeling of well-being, and the increase in appetite mostly due to steroid administration [10]. Many patients become obese during the first years after RT [11]. This causes an increase in the risk of developing arterial hypertension, cardiovascular disorders, new-onset diabetes mellitus, and hyperlipidemia [12]. All these factors lead to accelerated CAD [13]. Therefore, in renal transplant recipients, it seems crucial to implement as early as possible an effective nutrition program to achieve and maintain a correct body weight [14] with the final goal of improving graft survival. A further advantage associated with a healthy diet could be the reduction of the microinflammation that is commonly observed in transplant recipients [15]. Evidence is emerging, indeed, that dietary manipulation may beneficially influence inflammatory and even autoimmune diseases [16]. In particular, it has been suggested that the supplementation with anti-inflammatory n-3 polyunsaturated fatty acids (PUFAs) could exert beneficial effect in preserving the renal function in renal transplant recipients. On the contrary, n-6 PUFAs may act as a proinflammatory substance and may even promote kidney injury [17, 18].

Previous studies in renal transplant recipients focused on the effects of immunosuppressive drugs on body composition [19] or tried to identify the characteristics of patients at risk for excessive weight gain [20]. On the contrary, limited information is available on nutrient intake, and no study evaluated yet the intake of n-3 and n-6 PUFAs and their ratio in these patients. In addition, no data is available for the impact of the nutritional status on systemic inflammation and drug consumption in renal allograft recipients. Therefore, in the present study, we assessed dietary intake, with particular reference to n-3 and n-6 PUFA intake, in a cohort of long-term renal transplant patients, and we compared the results obtained with current nutrition recommendations. We also evaluated the association between nutritional, and inflammatory status and cardiovascular and antidiabetic drug assumption in these patients. 

## 2. Patients and Methods

### 2.1. Patients

A total of 96 patients were enrolled from September 2012 to December 2012 among the recipients of a renal transplant from a cadaver donor coming to the Nephrology Unit of the Federico II University of Naples as part of the periodic follow-up after transplantation for immunosuppressive drug monitoring and clinical and metabolic evaluation. Enrollment was performed according to the inclusion and exclusion criteria reported below and independently from the time elapsed from kidney transplantation.

 The mean transplant vintage was 7.2 ± 5.0 years. All the patients had a successful transplantation and none of them had to be subjected to a second organ transplant. Inclusion criteria were as follows: age >18 years, stable renal function in the last 3 months, absence of infection, cancer, or any acute illness in the last 2 months. The immunosuppressive regimen of all the patients included 4 mg/day of methylprednisolone plus cyclosporine (CYA) or tacrolimus (FK) and, in some patients, mycophenolate mofetil (MMF). Drug therapy was adjusted to reach trough CYA and FK blood levels of 150–200 and 6–10 mg/mL, respectively.

At the time of the enrollment, a comprehensive nutritional evaluation was performed as detailed here in after. A 7-day food diary was given to the patients who were instructed on how to fill it out at home. When the patients came back after a week to return the diary, a blood sample was collected for the determination of the biochemical markers of the nutritional and inflammatory status specified below. All the subjects gave their informed consent to the study that was approved by the Ethical Committee of the Medical School of the University Federico II of Naples.

### 2.2. Assessment of the Nutritional Status

The nutritional status of the patients was determined by measuring their body mass index (BMI), by assessing their body composition, and by evaluating specific biochemical nutritional markers in their plasma. 

BMI was calculated for each patient as the body mass divided by the square of height (kg/m^2^) [21]. According to the BMI classification of the World Health Organization, patients having a BMI between 18.5 and 24.9 were classified as *Normalweight* (NW), those with a BMI between 25.0 and 29.9 as *Overweight *(OW), and those with a BMI higher than or equal to 30 as *Obese *(OB). Waist to hip ratio (WHR) was measured as an indirect index of visceral obesity. WHR was measured by placing a measuring tape at the level of the umbilicus whereas the measuring tape was placed at the level of the widest part of the hip to measure hip circumference. Participants were considered centrally obese if WHR was 0.95 or over for male and 0.85 or over for female.

Body composition was assessed by bioelectrical impedance analysis (BIA). A tetrapolar 50 kHz bioelectrical impedance analyzer (BIA 101 RJL, Akern Bioresearch, Firenze, Italy) was used to measure resistance (R) and reactance (Xc). These parameters were converted by the software provided by the manufacturer in estimates of total body water (TBW), fat mass (FM), and fat free mass (FFM) [22, 23]. 

The following chemicals were assessed in peripheral blood samples as they are sensitive indicators of the nutritional status: blood urea nitrogen (BUN), creatinine, glucose, total cholesterol, high-density lipoprotein cholesterol (HDL-chol), low-density lipoprotein cholesterol (LDL-chol), triglycerides, albumin, calcium, and phosphorus. 

### 2.3. Dietary Assessment

Caloric intake and diet composition were determined through the analysis of a 7-day food diary that patients were required to fill out at home after each meal [24]. These data were examined by expert dietitians who calculated total daily intake and the percent contribution of micro- and macronutrients using validated food composition tables [25–27]. Total n-3 polyunsaturated fatty acids (n3-PUFAs) in the diet were calculated as the sum of the content in foods of eicosapentaenoic acid (EPA), docosapentaenoic acid (DPA), docosahexaenoic acid (DHA), and *α*-linolenic acid (ALA). Total n-6 polyunsaturated fatty acids (n6-PUFAs) were, instead, the sum of linoleic (LA) and arachidonic acid (AA). Although, using the food diary data, we assessed the average intake of sodium from foods, we could not estimate the amount of salt added to the food during cooking or at the table. Therefore, total sodium intake was estimated by evaluating urinary sodium excretion. 

The data obtained at the analysis of energy and nutrient intake were compared with the reference values reported in the guidelines of the Dietitian Association of Australia that still represent the only comprehensive dietary guidelines for kidney transplant patients [3]. In addition, we compared the data obtained in our patients with values recommended by K/DOQI guidelines for patients with stage I/II chronic kidney failure (CKD) [1, 2] that are often used in the clinics as the target for a correct nutritional status in kidney graft recipients. 

### 2.4. Analytical Procedures

BUN, creatinine, glucose, total cholesterol, HDL-chol, LDL-chol, triglycerides, albumin, calcium, and phosphorus were determined by standard analytical techniques. Serum high-sensibility C-reactive protein (hs-CRP) concentration was determined by a high-sensitivity ELISA assay (Bender MedSystems, Vienna, Austria). The lower detection limit was 3 pg/mL, and the overall intra-assay coefficient of variation has been calculated to be 6.9%. Interleukin 6 (IL-6) plasma concentrations were determined by ELISA using a commercially available kit (Quantikine; R&D Systems, Minneapolis, MN) as described elsewhere [28]. The lower detection limit of IL-6 assay was <0.70 pg/mL, and the coefficient of variation of both inter- and intra-assay was <5%. All samples were analyzed in duplicate.

### 2.5. Data Analysis

Data are expressed as mean ± SD. The statistical evaluation of the data was performed by Student's *t*-test for unpaired data in case of two group comparison or by one-way ANOVA followed by the post hoc Bonferroni's test in case of multiple group comparisons. Pearsons *χ* ^2^ test was used to compare prevalence among groups. The threshold for significance was set at *P* < 0.05. Data analysis was performed with the Statistical Package for Social Science software suite (IBM SPSS version 20.0) (IBM, New York, USA).

## 3. Results

A total of 96 kidney transplant recipients entered the study. The main demographic and anthropometric characteristics of these three groups of patients are reported in Table 1. Seventy-one percent of them were males. At the time of transplantation, 68.8% of patients had a normal BMI, 25.0% were overweight, and 6.3% obese. When the study was performed, approximately 7.2 ± 5.0 years after transplantation, these percentages had changed to the following: 52.1% normal weight (50 patients, 36 males and 14 females), 29.1% overweight (28 patients, 20 males and 8 females), and 18.8% obese (18 patients, 12 males and 6 females). A higher weight and BMI gain occurred in females compared to males during the years after kidney transplantation (overall, 19.8 ± 13.6% versus 6.4 ± 10.4% of pretransplant body weight, resp.; *P* < 0.001).

When we evaluated the body composition, we found that the percent of fat mass and the percent of free fat mass were significantly higher in OB than in OW and NW, and in OW than in NW patients. Sixty-eight percent of male and 64% of female patients showed higher WHR values than normal. When we examined the distribution of these patients across the three different BMI categories that we defined in Section 2, we found that WHR was higher than normal in 50% of male and 43% of female NW, in 90% of male and 75% of female OW, and in 83% of male and 100% of female OB. No significant difference was observed in extracellular water and TBW/FFM ratio, whereas body cell mass was higher in OB and OW than in NW patients (Table 1). There was no significant difference among the groups in the immunosuppressive regimen assumed by the patients. All patients were treated with 4 mg/day methylprednisolone plus tacrolimus (*n* = 38) or cyclosporine (*n* = 54) in addition to steroids. In 76 patients, the immunosuppressive protocol also included mofetil mycophenolate. There was no significant difference in the cumulative dose of steroids assumed by the patients of the three groups (Table 1). Two patients treated with CYA and none of those receiving FK experienced acute rejection episodes during the study. The percentage of patients assuming lipid-lowering or antidiabetic drugs was significantly higher in OB and OW than in the NW groups (78%, 71%, and 52%; 4%, 14%, and 22%; resp.).

Table 2 shows daily energy and nutrient intake of the 3 groups of patients (by BMI category) compared to dietary reference intakes. Total energy intake was significantly higher in OB than in NW, but in all the groups both energy and protein intake, however expressed, were higher than recommended by guidelines [1, 3, 5, 7–9]. Carbohydrates (CHO) intake, conversely, was in the suggested range [4, 5, 8, 9] in all the groups, although a significant difference was observed between NW and OW (53.8 ± 4.2%, versus 50.3 ± 6.1% of total energy intake, *P* < 0.05, ANOVA). 

In all the BMI groups, total fat intake was close to that recommended by guidelines [1–5, 7–9]. However, it was higher in OW and OB groups compared to NW group. Relevant discrepancies from guidelines were observed in fatty acid composition of the diet: in fact, in all the BMI groups, saturated fatty acids intake was higher, while monounsaturated and polyunsaturated fatty acids intake was lower than the recommended intake [1–5, 7–9]. The intake of saturated fatty acids was higher in OB and OW than in NW (*P* < 0.05 OW versus NW). Concerning the intake of polyunsaturated fatty acids, in all patients n-6 and n-3 PUFA intakes were both lower than the recommended values [3, 6] with a ratio n-6/n-3 being remarkably high in all the groups (>5.4) and particularly in OB group (6.38 ± 2.3). A positive correlation between weight gain and both fat (*r* = 0.28; *P* < 0.005) and energy (*r* = 0.25; *P* < 0.013) intake was observed.

Although the average intake of sodium from foods was assessed, it was not possible to measure the amount of salt added to the food during cooking or at the table. However, total sodium intake was estimated from urinary sodium excretion. Total salt intake was high [3–5, 8], exceeding 10 g/day in all groups. Dietary fiber intake was lower than those recommended by guidelines [2–4, 8, 9] in all groups, and particularly in NW and OW groups. Dietary intakes of calcium and phosphorus resulted lower than the recommended intake [3, 7, 8] in all groups. 

Table 3 illustrates some biochemical parameters of the groups under study. Noticeably, OB patients showed higher signs of systemic inflammation. Indeed, circulating levels of IL-6 and hs-CRP were significantly higher in the OB than in the NW group. Moreover, serum hs-CRP was positively correlated with WHR (*r* = 0.26; *P* = 0.012). All the groups showed high triglycerides plasma levels according to the National Cholesterol Education Program (NCEP) Adults Treatment Panel III (ATP III) criteria [4], but no further difference was observed in other parameters (Table 3). Because it has been reported that plasma lipoprotein profile and glycaemia are differentially affected by CYA and FK [29], we compared these laboratory parameters in patients of our series assuming these two different drugs. The only significant difference was in LDL-cholesterol plasma level that was significantly lower in CYA-treated patients. However, significantly, more patients were also assuming lipid-lowering and antidiabetic drugs in the subgroup of subjects receiving CYA than in those treated with FK (67% versus 53%, and 16% versus 7%, resp.).

## 4. Discussion

The main finding of the present work is that in long-term renal transplant patients, dietary intake differs from guideline recommendations because of a higher-energy and saturated fat intake, a lower unsaturated, n-6, and n-3 PUFA, and fiber consumption. These discrepancies from nutritional recommendations are associated with a higher prevalence of obesity, with high systemic inflammation, and with a higher assumption of cardiovascular, lipid-lowering, and antidiabetic drugs.

At the time of the study, approximately 7 years after transplantation, mean body weight of our patients was 10.3% higher than pretransplant values. The prevalence of obesity in our patient population was 18.8% consistent with previous reports [30]. Our study shows that the prevalence of obesity in the renal transplant population is higher than in general population (approximately 11% in Italy) [31]. Previous studies suggested that the high prevalence of obesity after kidney transplantation may depend on an excessive food intake. This may be caused in renal allograft recipients by different factors including the increased appetite induced by glucocorticoids, the increased feeling of well-being, and the fact that patients stop assuming the strict dialysis diet to switch to an unrestricted diet. In keeping with this hypothesis, we found that in our patients the energy intake exceeded the values recommended by current nutritional guidelines (25 to 30 kcal/kg/day) [1, 3, 5, 7–9]. Moreover, we observed a strong correlation between total energy intake and the increase in body weight that occurred during the years after transplantation before the beginning of the study. These data further support the idea that excessive energy intake contribute to obesity after renal graft. In our renal transplant recipients, most of fat accumulation that occurs after kidney transplantation seems to be of the abdominal type as indicated by the high prevalence of patients with higher than normal WHR values. The evidence that visceral fat accumulates in these patients could explain the close link between obesity and inflammation that we found in our study. Interestingly, not only hs-CRP and IL-6 concentrations were higher in the OB subgroup but also a significant correlation was found between WHR and hs-CRP. This suggests that the accumulation of fat in general and especially of visceral fat could have a role in causing systemic microinflammation in kidney transplant recipients. This hypothesis is in agreement with previous data from our group showing that visceral fat contributes to increased plasma IL-6 and CRP in obesity [32]. Importantly, both IL-6 and CRP are strong predictors of all-cause and cause-specific cardiovascular mortality and they can, at least in part account for the well-established association between obesity, as defined on the basis of BMI, and high blood pressure, and unfavorable lipid and glucose metabolism profile [33]. Interestingly, WHR seems to be a stronger predictor of cardiovascular risk than BMI [34]. Under this respect, it is worth mentioning that almost half of our NW patients (50% of males and 43% of females) had a higher than normal WHR and, therefore, they showed a “normal weight obesity”. This nosological entity has been recently introduced to describe those patients that have a normal BMI despite a higher than normal body fat content. Importantly, normal weight obesity is associated with cardiovascular risk factors and also with increased mortality [35–38]. Therefore, our findings suggest that lifestyle and nutritional interventions could be helpful in kidney transplant recipients even in the absence of overt obesity when WHR is higher than normal. Such an approach could lead to a decrease in systemic inflammation and, possibly, improve long-term prognosis. Further studies on a larger patient population evaluated for a longer time will be necessary to strengthen this hypothesis. 

Little attention has been given in previous studies to the different contribution of different nutrients to the increase in energy intake in long-term transplant patients. In the present study, we addressed this issue because it could have important implications both in weight gain and in the risk of cardiovascular complications and CAD. Although mean total fat intake in our patients was within guideline recommendations, there was a strong correlation between total fat intake and the weight gain. The role of dietary fat intake in the development of obesity after kidney transplantation has not been studied before. Several studies evaluated its involvement in the genesis of obesity in general population yielding to controversial results. Indeed, some cross-sectional studies found no association [39], whereas others reported a positive association [40] between high-fat intake and obesity. The results of the present study stand for a positive relationship between fat intake and weigh it gain. Several factors could account for this association. First, fat is the most energy dense macronutrient. Second, fat provides a lower satiety feeling, and its great flavor and palatability may lead to a greater consumption of fatty foods. Dietary fat intake composition in our patients differed from guidelines because the content in saturated fat was higher and that in monounsaturated and polyunsaturated fats was lower than recommended. Moreover, the n-6/n-3PUFAs dietary ratio was higher than current recommendations in all patients. The excess in saturated fats coupled with the high n-6/n-3PUFAs dietary ratio provides an additional risk for development of cardiovascular diseases [41]. In addition, the association between high n-6/n-3PUFAs dietary ratio and obesity may account for the high IL-6 and hs-CRP that we found in our OB patients [42]. These laboratory findings are an index of systemic microinflammation, a condition occurring in obesity where it contributes to increase cardiovascular risk [43]. Importantly, systemic microinflammation is an auto-maintaining process because it promotes further weight gain that further worsen systemic inflammation [43, 44]. 

 Another important difference that we found between the nutrient composition of the diet of our long-term kidney transplant recipients and that recommended in the guideline was the higher content in protein intake. This could be relevant as a causative factor for CAD. Indeed, high protein intake may contribute to renal allograft injury [45] whereas a correct intake of proteins may delay the course of chronic allograft dysfunction, by reducing glomerular hyperfiltration [46]. 

Average CHO intake is close to that recommended in Guidelines [4, 5, 8, 9] in all patients; we cannot exclude, however, that obese patients have deliberately underreported their real CHO intake [47]. 

Although we could not accurately measure total sodium intake from dietary records, values estimated from the measurement of sodium urinary excretion stand for an intake substantially higher than recommended. This can be due to the impaired ability of patients with kidney disease to detect or taste salt in food [48] that makes them add much more sodium than normal to improve food palatability. In transplanted patients, dietary sodium may have important effects on proteinuria, control of hypertension, and maintenance of an optimal volume status [49]. 

Finally, we found that daily dietary fiber intake in our long-term renal allograft recipients was significantly lower than recommended in guidelines [2–5, 8, 9, 50]. The low-level fiber consumption coupled with high-energy intake also put these patients at risk for gaining weight or developing obesity [51]. Several studies showed that a high fiber intake prevents inflammation, metabolic syndrome, and cardiovascular diseases, which are highly prevalent after RT [52].

The hypothesis that the obesity-associated discrepancies in nutrient intake from recommendations in guidelines could have a role in worsening the cardiovascular risk of these subjects was supported by the evidence that the percent of cardiovascular, antidiabetic, or lipid-lowering drug assumption was higher in OB and OW than in NW patients.

The major limitation of our study is that we compared the data on nutrient intake that we obtained from our patients with those contained in guidelines that were not specifically drawn for renal allograft recipients. Such guidelines, indeed, are not available yet with the only exception of those of the Dietitian Association of Australia in 2008 [3], and in clinical practice the guidelines usually used are those for stage I/II chronic kidney failure. The results of our study showing the association between body weight gain, systemic inflammation, and the use of multiple drugs in kidney transplant recipients emphasize the need to plan further studies with the aim of releasing official guidelines for nutritional treatment of these patients. Another important limitation is that our patients were all from southern Italy, and their dietary habits could reflect regional variations and, therefore, not be representative of the general behavior of kidney transplant recipients.

In conclusion, we showed that nutrient composition in long-term renal allograft recipients differs from recommended values because of a higher protein, sodium, and saturated fat and of a lower fiber and n-3 and n-6 polyunsaturated fat content. These discrepancies from guidelines are associated with obesity, systemic inflammation, and higher drug consumption.

## Figures and Tables

**Table 1:** Demographic, anthropometric, and body composition data and the pharmacological of treatment the three groups of kidney transplant recipients divided according to BMI.

	NW *n* = 50	OW *n* = 28	OB *n* = 18
Male/female	36/14	20/8	12/6
Age, years	51.5 ± 10.4	48.7 ± 8.5	50.3 ± 10.1
Transplant time, years	7.8 ± 5.1	5.6 ± 4.3	7.9 ± 5.5
Pretransplant BW, Kg	58.2 ± 12.0	66.6 ± 9.4*	74.4 ± 12.1*
Pretransplant BMI, Kg/m²	21.6 ± 2.9	24.2 ± 2.3*	28.2 ± 3.2^∗*∧*^
BW, Kg	60.5 ± 9.3	74.5 ± 36.7*	87.7 ± 11.7^∗*∧*^
BMI, Kg/m²	22.5 ± 2.0	27.2 ± 1.3*	33.3 ± 3.2^∗*∧*^
ΔBW, % of pre-transplant BW	5.6 ± 11.8	13.0 ± 11.5*	19.0 ± 12.6*
TBW, % BW	59.4 ± 6.3	54.8 ± 4.9*	47.9 ± 3.8^∗*∧*^
ECW, % TBW	45.6 ± 3.9	43.9 ± 4.8	44.2 ± 4.4
FM, % BW	19.9 ± 8.7	25.5 ± 6.4*	35.6 ± 6.7^∗*∧*^
FFM, % BW	80.0 ± 8.7	74.4 ± 6.4*	64.4 ± 6.7^∗*∧*^
BCM, Kg	25.4 ± 5.8	30.0 ± 6.1*	29.5 ± 5.7*
TBW/FFM	0.74 ± 0.02	0.73 ± 0.01	0.74 ± 0.02
Systolic blood pressure (mmHg)	126 ± 11	130 ± 14	128 ± 8
Diastolic blood pressure (mmHg)	80 ± 7	81 ± 3	80 ± 5
Cumulative corticosteroid dose (g)	10.8 ± 8.5	8.1 ± 6.4	8.9 ± 8.1
Oral antidiabetic drugs/Insulin therapy (%)	0/4	14/0	0/22
Lipid-lowering therapy (%)	52	71	78
Antihypertensive therapy (%)	100	100	100

BW: body weight; BMI: body mass index; FM: fat mass; FFM: fat-free mass; TBW: total body water; ECW: extracellular water; BCM: body cell mass.

*P < 0.05 versus NW; ^*∧*^ *P* < 0.05 versus OW; ANOVA (with Bonferroni's posttest).

**Table 2:** Daily energy and nutrient intake of the 3 groups of patients (by BMI category) compared to the recommended daily intake (DRI). NW: normal weight, OW: overweight, OB: obese.

	NW *n* = 50	OW *n* = 28	OB *n* = 18	DRI^*∧*^
Energy intake (Kcal/day)	1995 ± 282	2126 ± 264	2216 ± 358*	—
Energy intake (Kcal/Kg°/day)	32.9 ± 4.9	33.9 ± 4.0	35.5 ± 6.8	25–30^a,c,e,g,h,i^
Protein^#^ (% kcal/day)	15.4 ± 1.4	15.7 ± 2.6	15.2 ± 1.0	—
Protein^#^ (g/Kg°/day)	1.27 ± 0.2	1.32 ± 0.2	1.38 ± 0.2	0.8–1.0^a,c,e,g,h,i^
Protein^§^ (g/Kg°)	1.20 ± 0.2	1.25 ± 0.2	1.29 ± 0.3	—
Carbohydrates (% kcal/day)	53.8 ± 4.2	50.3 ± 6.1*	51.6 ± 4.7	50–60^d,e,h,i^
Total Fat (% kcal)	28.9 ± 3.4	31.9 ± 6.5**	31.3 ± 2.8*	25–35^a,b,c,d,e,g,i^; <30^h^;
Saturated fat (% kcal/day)	7.8 ± 1.3	8.9 ± 1.9*	8.2 ± 1.3	<7^a,d,i^; <8^c^; <10^g,h^
Monounsaturated fat (% kcal/day)	16.1 ± 2.1	17.5 ± 3.5	17.3 ± 1.9	>20^b,c,d,i^; 10–15^h^
Polyunsaturated fat (% kcal/day)	3.2 ± 0.2	3.6 ± 0.9**	3.7 ± 0.7**	>10^b,d,h,i^
Omega 3 (% kcal/day)	0.50 ± 0.1	0.57 ± 0.1*	0.52 ± 0.1	—
Omega 3 (g/day)	1.12 ± 0.2	1.34 ± 0.4**	1.27 ± 0.3	2-3^c,f^
Omega 6 (% kcal/day)	2.67 ± 0.3	3.00 ± 0.9	3.15 ± 0.7*	8–10^c^
Omega 6 (g/day)	5.94 ± 1.3	7.12 ± 2.5*	7.89 ± 2.7**	10^f^
Omega 6/Omega 3	5.41 ± 1.3	5.62 ± 2.1	6.38 ± 2.3	1/1 to 4/1^j^
Fiber (g/day)	21.3 ± 5.9	20.8 ± 5.5	25.1 ± 8.3	20–30^b,c,d,h,i^
Cholesterol intake (mg/day)	169.5 ± 55.9	185.2 ± 49.1	171.7 ± 76.8	<200^b,d^; <300^h^
Food sodium intake^#^ (mg/day)	1750 ± 601	1892 ± 559	2171 ± 671	—
Total NaCl intake^§^ (g/day)	10.3 ± 4.2	11.3 ± 3.4	12.7 ± 3.1	6–8^c,e,h^; <6^d,k^
Calcium (mg/day)	663 ± 170	747 ± 207	710 ± 158	800–1500^c,g,h^
Phosphorus (mg/day)	1112 ± 242	1237 ± 247	1188 ± 212	1200–1500^g,h^

°kg of ideal weight; ^#^estimated by diet record;   ^§^evaluated by urinary excretion of NaCl or Urea; ^*∧*^DRI: dietary reference intakes; *P < 0.05 versus NW, **P < 0.01 versus NW.

^a^[1], ^b^[2], ^c^[3], ^d^[4], ^e^[5], ^f^[6], ^g^[7], ^h^[8], ^i^[9], ^j^[53] ^k^[25].

**Table 3:** Biochemical parameters and inflammatory markers (hs-CRP and IL-6) in the three groups of kidney transplant recipients (NW: normal weight, OW: overweight, OB: obese).

	NW *n* = 50	OW *n* = 28	OB *n* = 18
eGFR° (mL/min)	50.8 ± 19.4	54.1 ± 13.2	63.0 ± 25.2
Plasma glucose (mg/dL)	77.7 ± 9.1	81.3 ± 15.0	82.9 ± 17.6
Plasma total cholesterol (mg/dL)	188.9 ± 33.5	181.0 ± 25.2	185.9 ± 31.4
Plasma HDL cholesterol (mg/dL)	49.4 ± 11.7	55.0 ± 14.9	50.0 ± 19.5
Plasma LDL cholesterol (mg/dL)	107.5 ± 34.9	96.3 ± 19.9	105.4 ± 30.4
Plasma tryglycerides (mg/dL)	153.5 ± 74.2	157.0 ± 46.8	154.3 ± 63.0
Serum uric acid	6.2 ± 0.7	6.3 ± 1.1	5.9 ± 1.0
Calcium (mg/dL)	9.7 ± 0.5	9.9 ± 0.4	9.6 ± 0.2
Phosphorus (mg/dL)	3.4 ± 0.8	3.2 ± 0.4	3.3 ± 0.4
Albumin (g/dL)	4.4 ± 0.3	4.5 ± 0.2	4.5 ± 0.3
hs-CRP (mg/L)	2.7 ± 2.4	4.0 ± 3.7	4.8 ± 3.9*
IL-6 (pg/mL)	4.5 ± 3.2	5.0 ± 4.4	7.3 ± 5.0*
Fibrinogen (mg/dL)	377.1 ± 123.4	326.0 ± 45.7	372.9 ± 101.1

°Glomerular filtration rate (expressed by MDRD formula).

*P < 0.05 versus NW.
